# Editorial: Small cell lung cancer: New drugs and strategies

**DOI:** 10.3389/fmed.2023.1140642

**Published:** 2023-01-26

**Authors:** Diego L. Cortinovis, Alessandro Morabito

**Affiliations:** ^1^SC Medical Oncology, Fondazione IRCCS San Gerardo dei Tintori, Monza, Italy; ^2^Medical Oncology, Thoracic Department, Istituto Nazionale Tumori “Fondazione G. Pascale”-IRCCS, Naples, Italy

**Keywords:** SCLC, immunotherapy, lurbinectedin, anti-DLL3 drugs, Aurora kinase inhibitors

Small Cell Lung Cancer (SCLC) is an aggressive disease with a dismal prognosis at 5 years (1). After decades of nihilism, immune-check point inhibitors combined with chemotherapy led to a new standard first line treatment improving the overall survival rate and increasing also the number of the so-called long-survivors (2). A way that may lead to an improvement in recognizing some “Achille heels” of SCLC is to understand the biological differences in a disease considered so far like a monolith. The right direction could be the new proposal of classification that takes into account the different expressions of key transcription regulators like ASCL1-high, NEUROD1-high, POU2F3-high, and YAP1-high. This effort to categorize SCLC in different subgroups may lead to a different way to build therapeutic strategies and currently prospective trials to define the usefulness of this classification are ongoing (3). Despite the huge progress achieved in the NSCLC counterpart related to the discovery of response predictive biomarkers, these remain relatively unknown in SCLC, making personalized medicine for this malignancy still a chimera (4).

The main aim of our Research Topic is to explore new drugs and strategies in the field of SCLC, given the importance of summarizing some points related to the innovations that have emerged from the most recent clinical research (5). In particular, this issue includes fourteen articles focusing on original research (5 papers), reviewing some aspects of therapeutic strategy (7 papers), and 2 case reports to accompany the reader through all the aspects that distinguish the SCLC complex world, building a bridge between the present and future of the clinical management of this cancer.

Our Research Topic starts from the little-explored world of surgical management of early-stage SCLC, in which the risk-benefit balance of the surgical approach is still debated. In the review presented by Petrella et al., the role of surgery is reviewed in the light of literature data and the personal experience of the authors. Stage I SCLC is a really rare entity, mostly diagnosed incidentally: however, even if the rate of surgical resection remains low (1 to 6% in limited disease) lobectomy with radical lymphadenectomy is considered the gold standard surgical procedure, leading the overall survival at 5 years in nearly 50% of the patients who underwent the surgical approach. The monocentric experience reported in this paper underlines that patients with stage I pathological SCLC had a 76% of 5 years overall survival. This excellent prognosis is certainly guided by several prognostic factors including the absence of positive lymph nodes and the low diameter of the tumor. The clinical impact of the number of lymph nodes dissected (LNDs) on overall survival in N0 SCLC was assessed by Takamori et al. who queried the National Cancer Database (NCDB) exploring patients with very early SCLC (stage I-II as AJCC 7^th^ edition) treated with a lobectomy between 2004 and 2017. They reported for the first time that SCLC patients with ≥3 LNDs had a significantly longer OS than those with <3 LNDs. The multivariate analysis confirmed that ≥3 LNDs was an independent predictor for OS. In both publications the surgical approach appears feasible and recommended particularly in stage I SCLC: however, to better define this population, an adequate lymph node sampling is of fundamental importance to consider the surgical intervention oncologically complete, while the number of lymph nodes removed remains a surrogate of the lymph node pathological situation, distinguishing the population of true N0 patients who have a decidedly excellent prognosis.

The main part of our Research Topic is dedicated to stage IV SCLC which affects more than 80% of diagnosed cases. One of the major fields of interest is related to the search for prognostic and predictive factors of response to treatments, including chemotherapy, immunotherapy or new drugs. Zhou et al. in their systematic review investigated the prognostic value of the systemic immune-inflammation index (SII) for SCLC. A set of bio humoral factors that are easy to use would be of importance to better evaluate patients to be referred to first-line treatment and to reduce costs and turnaround time for extensive, massive deep gene panel testing. SII, as reported in their paper, consists of a set of biomarkers including neutrophil-to-lymphocyte ratio, platelet-to-lymphocyte ratio, C-reactive protein/albumin ratio, that had a prognostic role in a series of different malignant tumors. The authors concluded that also in advanced SCLC this composite biomarker tool had a relationship with prognosis and could be useful to indicate the best strategy for each patient. The role of new biomarkers for predicting the activity of immunotherapy is welcome and in the original research reported by Tang et al. C-C Motif Chemokine Ligand 5 (CCL5) expression on tumoral micro-environment has been extensively studied in a cohort of SCLC patients treated with immune-checkpoint inhibitors (ICIs). The authors found that CCL5 high expression correlated positively with overall survival and its level of expression is associated with the co-expression of other immune-checkpoint proteins like PD1/PDL1, CTLA4 among others; although its role could be further clarified in prospective trials, there are some clues about a possible role as a predictive biomarker in patients treated with ICI+DNA damage agent (PARP inhibitor). Another fascinating way to predict the efficacy of chemotherapy is to study chemosensitivity in circulating tumoral cells (CTCs). This is the main topic of the original research reported by Ju et al.. In their paper, the authors showed the results of a retrospective study conducted on SCLC patients treated with different lines of chemotherapy: they tested the susceptibility to 6 different chemotherapeutic agents monitoring CTC counts and collecting them. The reduction of CTC counts correlated positively with therapy response. Unfortunately, the administration of a newer chemotherapy line to SCLC patients based on the drug susceptibility test of CTCs failed to demonstrate a clinical activity: the weakness of a very limited sample size does not allow to draw a definitive conclusion about this experimental procedure.

Following the recent therapeutic innovations in first-line therapies and the emergence of potentially useful new drugs, the other part of our Research Topic is fully dedicated to new therapeutic strategies. Belluomini et al. extensively reviewed the available literature data about SCLC management, with a particular focus on special populations such as elderly or low-performance status patients (ECOG PS 2). This aspect has been particularly dealt with in the literature review conducted by Giunta et al. that underline the evidence and weaknesses of the first line strategy with the modern combination with CT+ICIs. The discrepancies and the difference between clinical trials results and the real-world evidence (RWE) are depicted by Rittberg et al. who described in their original research how the majority of the patients in a Canadian retrospective cohort analysis did not have the clinical characteristic to receive the triple first-line combination in the first line setting claiming the need to better understand which strategy may be really conducted in RWE.

The hopes regarding the new therapeutic strategies are entrusted also to new drugs with different mechanisms of action compared to classic chemotherapeutic agents and ICIs: in the papers of Manzo et al. and Cortinovis et al. all the findings about lurbinectedin and anti-DLL3 agents were exploited, while a focus on Aurora kinase inhibitor was extensively reviewed in the paper of Stefani et al.. SCLC is also hard to treat due to the presence of particular syndromes such as paraneoplastic syndromes that accompany its diagnosis. Ectopic Cushing's syndrome was addressed by Piasecka et al. who reviewed monocentric SCLC medical records, showing that almost 12% of the population could present with this syndrome which remains potentially underdiagnosed. Finally, some peculiar clinical aspects are presented in 2 clinical cases reported by Wang et al. and Zhang et al. about a rare phenotype switching from SCLC to NSCLC and a clinical case with a long survival due to a personalized therapeutic strategy.

In summary, new drugs and strategies will improve the prognosis of this orphan disease, but several challenges remain in the management of SCLC, including the lack the true predictive biomarkers to address the right population to newer therapeutic strategies, the lack of information regarding special populations excluded by clinical trials, the need of more insights about RWE, decreasing the gaps between clinical practice and research. We hope that this Research Topic will be of interest for the reader suggesting new ideas for future research.

## Author contributions

DLC and AM: conceptualization, writing—original draft preparation, writing—review and editing, and supervision. All the authors have read and agreed to the published version of the manuscript.
